# Incidence Rates of RSV‐Associated Hospitalizations Among Adults in Middle Tennessee, United States, October 2022 Through September 2023

**DOI:** 10.1111/irv.70150

**Published:** 2025-08-25

**Authors:** Carlos G. Grijalva, Jesse O. Wrenn, Jonathan E. Schmitz, Karen F. Miller, Adrienne Baughman, Ian D. Jones, James D. Chappell, Natasha B. Halasa, Paul W. Blair, Yuwei Zhu, H. Keipp Talbot, Jonathan D. Casey, Fatimah S. Dawood, Diya Surie, Wesley H. Self, for the Investigating Respiratory Viruses in the Acutely Ill (IVY) Network

**Affiliations:** ^1^ Vanderbilt University Medical Center Nashville Tennessee USA; ^2^ Centers for Disease Control and Prevention Atlanta Georgia USA

**Keywords:** adults, hospitalization, rates, respiratory syncytial virus

## Abstract

We estimated the burden of RSV‐associated hospitalizations in US adults 1 year prior to RSV vaccine introduction. The overall annual incidence rate of RSV‐associated hospitalization was 31.47 (95% CI: 21.89–43.97) per 100,000 adults. Rates were 10‐fold and 17‐fold higher among adults 60 to 74 years and ≥ 75 years compared with adults 18 to 49 years old. This prospective assessment demonstrated the burden of RSV‐associated hospitalizations among adults, with the highest hospitalization rates among adults ≥ 60 years old, in the year prior to RSV vaccine introduction.

## 1 Introduction

In 2023, the Centers for Disease Control and Prevention (CDC) recommended respiratory syncytial virus (RSV) vaccination for older adults. As of June 2024, RSV vaccination is recommended in the United States for adults ≥ 75 years old and those 60 to 74 years old at increased risk for severe RSV disease [1, 2].

Ascertaining the impact of the RSV adult vaccination program is of public health interest and requires contemporary assessments of RSV burden preceding vaccine introduction [3]. We conducted a prospective study to estimate the burden of RSV‐associated hospitalizations in adults during the year prior to RSV vaccine introduction in the United States.

## 2 Methods

From October 1, 2022, through September 30, 2023, we sought to identify all RSV‐associated hospitalizations at Vanderbilt University Medical Center (the “surveillance hospital”) in Nashville, Tennessee, from adults residing in a nine‐county catchment area. The surveillance hospital is a quaternary care teaching hospital licensed for acute and specialty care. It provides care for a sizable fraction of the population in middle Tennessee and surrounding counties and states. We identified RSV‐associated hospitalizations via study‐dedicated “research” testing of preserved residual specimens from the ongoing COVID‐19 screening program at the surveillance hospital and routine clinical RSV testing.

### 2.1 RSV Research Testing

Per hospital COVID‐19 screening policy, nasal swabs were collected from all hospitalized patients upon hospital admission and tested for SARS‐CoV‐2 in a hospital laboratory through April 11, 2023. After that date, specimens were collected from all patients hospitalized with respiratory symptoms. For this study, preserved frozen residual aliquots of nasal swab specimens collected during the COVID‐19 screening program were retrieved by the study team and tested for RSV in a research laboratory using quantitative RT‐PCR with the Hologic Panther FluA/FluB/RSV assay. Results of RSV testing from these specimens are reported in this study as “research test results.”

### 2.2 RSV Clinical Testing

Throughout the study period, clinicians ordered RSV testing according to routine clinical care based on their clinical discretion. These clinical tests were conducted by RT‐PCR on nasal swab specimens collected by clinical personnel. Results of RSV testing from routine clinical care completed during the acute hospitalization are reported as “clinical test results” in this study.

### 2.3 Ethical Considerations

This activity was reviewed by the CDC and the institutional review board at Vanderbilt University Medical Center, deemed public health surveillance with a waiver of participant informed consent, and was conducted consistent with applicable federal law and CDC policy (45 C.F.R. part 46.102(l)(2), 21 C.F.R. part 56; 42 U.S.C. §241(d); 5 U.S.C. §552a; 44 U.S.C. §3501 et seq).

### 2.4 Hospitalizations for RSV

For hospitalizations without research or clinical RSV testing, we projected the probability of RSV detection. These projections accounted for patient age, time period, and whether the patient had a discharge diagnosis for acute respiratory illness (ARI) or exacerbation of a cardiopulmonary condition. The age categories were: 18–49 years; 50–59 years; 60–74 years; and ≥ 75 years. These age categories were selected based on the current CDC RSV vaccine recommendations, which provide different guidance for adults < 60 years old, 60–74 years old, and ≥ 75 years old.

The 1‐year study was divided into three different time periods based on the timing of the change in COVID‐19 screening practices at the surveillance hospital (and hence the availability of residual nasal swab specimens for RSV research testing) and local RSV activity (which showed very low circulation during the summer months). We combined these factors to define three mutually exclusive time periods: October 1, 2022 to April 11, 2023; April 12, 2023 to July 31, 2023; August 1, 2023 to September 30, 2023.

For each age group during each of the three periods, we first summarized the number of hospitalizations with clinical RSV tests, the number of hospitalizations with and without an ARI or exacerbation of a cardiopulmonary condition discharge diagnosis with research RSV tests done, and the number of hospitalizations with and without an ARI or exacerbation of a cardiopulmonary condition discharge diagnosis (Table S1) without clinical or research RSV tests done. For hospitalizations with and without ARI or exacerbation of a cardiopulmonary condition discharge diagnoses and research RSV tests done, we computed the proportion of RSV detections and applied these estimated proportions to the corresponding hospitalizations without clinical or research tests done to estimate the projected number of RSV hospitalizations in each group. These calculations were done separately for each period and age group and then added up to calculate the estimated totals for each group. Then, the probability of RSV detection at the surveillance hospital for each age group was calculated by dividing the corresponding observed number of RSV cases (including clinical and research testing) by the estimated total number of RSV cases (i.e., observed plus projected cases) (Tables S2–S6).

### 2.5 Market Share of Surveillance Hospital for Catchment Area Hospitalizations

Hospitalizations included in this study were limited to those by residents from a nine‐county catchment area in Middle Tennessee surrounding the surveillance hospital [4, 5]. We used available data supplied from the Tennessee Hospital Discharge Data System from the Department of Health to calculate age‐group–specific proportions of all‐cause hospitalizations among catchment area adults that occurred at the surveillance hospital (“market share”) in 2022 (Table S7).

### 2.6 Incidence Rate Calculations

To estimate the total number of RSV hospitalizations in the catchment area for each age group, we weighted the observed RSV hospitalizations from the surveillance hospital via clinical and research testing by the product of the probability of RSV detection at the hospital and the probability of hospitalization at the surveillance hospital (i.e., market share). We estimated the annual incidence of RSV hospitalizations for each group by dividing the estimated number of RSV hospitalizations by the corresponding population estimates derived from the US census for midyear 2023 (Table S8). To estimate the 95% confidence interval for the number of RSV hospitalizations in the catchment area, we used a bootstrap approach. This method involved resampling the data multiple times to assess variability and uncertainty derived from the probability of RSV detections and market share estimates. We created 1000 bootstrap samples for RSV detections and 1000 bootstrap samples for market share estimates by randomly resampling the data with replacement [6]. For each set of samples, we recalculated the estimated RSV hospitalizations. The 2.5th and 97.5th percentiles of these 1000 estimates provided the lower and upper bounds for the 95% confidence interval for RSV hospitalizations in the catchment area. Census estimates were considered fixed and thus without variance. Annual incidence rates and their 95% confidence intervals were expressed as RSV‐associated hospitalizations per 100,000 population. Rate ratios and their 95% confidence intervals were derived from estimates using the Method of Variance Estimates Recovery for a ratio (Table S9) [7].

### 2.7 Secondary Analysis

Since some studies have suggested that RT‐PCR of single respiratory specimens may have imperfect sensitivity for detecting RSV infections in adults when compared with a composite of paired serology, sputum, nasopharyngeal, and nasal swabs, we conducted a separate scenario set of estimates assuming the sensitivity of RSV detections in this study (based on nasal swabs) was 66%. This secondary scenario analysis was achieved by multiplying observed RSV detections in the primary analysis by (1/0.66) (Table S9) [4, 8].

### 2.8 Examination of RSV Hospitalizations Detected by Research Testing and Without ARI

There were 15 RSV‐associated hospitalizations detected only by research testing and without recorded diagnoses of ARI or exacerbations of cardiopulmonary conditions. Most of these detections occurred during the period of widespread COVID screening. Five (33.3%) of these detections occurred among adults aged 60 to 74 years, whereas only three (20%) occurred among adults aged 75 years or older (Table S10).

## 3 Results

During the 1‐year study, there were 21,392 hospitalizations at the surveillance hospital among catchment area adults. RSV testing was completed for 9,958/21,392 (46.6%) hospitalizations; 49/9,958 (0.49%) had an RSV detection, including 15 detections through research testing and without study ARI‐related diagnoses (Figure S1).

**FIGURE 1:**
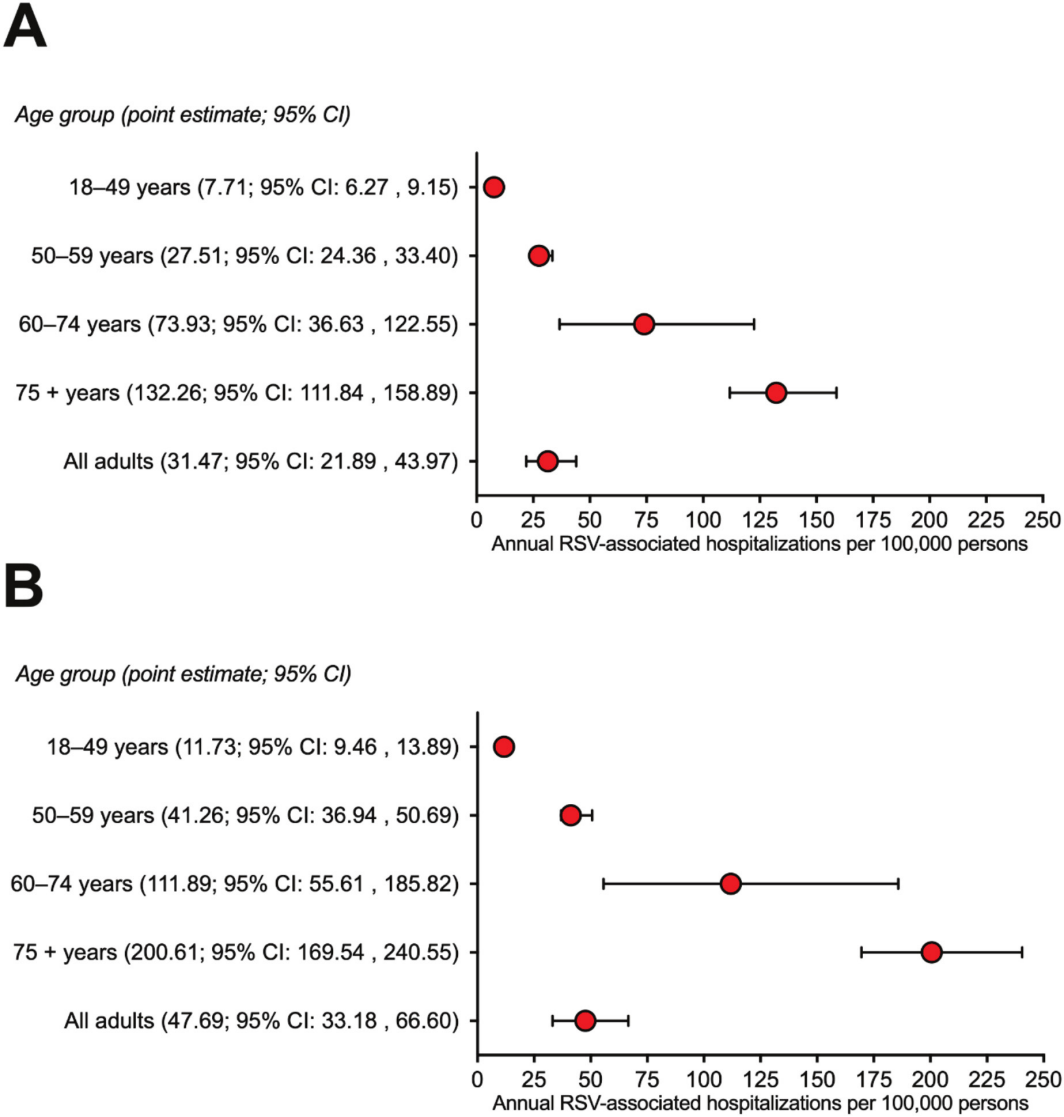
Estimated incidence rates of RSV‐associated hospitalizations per 100,000 adults in Middle Tennessee (nine counties), USA, October 2022 through September 2023 (the 12 months prior to introduction of RSV vaccines in the United States). The primary analysis (Panel A) did not adjust for potentially imperfect sensitivity of RT‐PCR testing of nasal swabs for RSV detection. The secondary analysis (Panel B) assumed that the study RSV detections, based on RT‐PCR testing of nasal swabs, had a sensitivity of 66%.

After accounting for the untested population and the surveillance hospital market share, the overall annual incidence rate of RSV‐associated hospitalization was 31.47 (95% CI: 21.89 to 43.97) per 100,000 adults. Rates were 10‐fold and 17‐fold higher among adults aged 60–74 years and ≥ 75 years compared with adults 18–49 years old (Figure 1A). Estimated rates that accounted for imperfect detection sensitivity were slightly higher (Figure 1B).

## 4 Discussion

In the year preceding the introduction of RSV vaccination in the United States, RSV was associated with a substantial burden of adult hospitalizations, especially among adults ≥ 60 years old, underscoring the potential benefits of RSV vaccination in older adults. Overall and age‐specific incidence rate estimates are consistent with previous estimates from prospective studies conducted before and during the COVID‐19 pandemic [4, 5, 9]. These estimates provide a reference for evaluations of the RSV vaccination program in subsequent seasons.

Extrapolating our RSV hospitalization rates estimates to the US adult population, there were approximately 82,000 RSV‐associated hospitalizations in the year preceding vaccine introduction. For general comparison with the burden of other vaccine preventable respiratory diseases, for the same study period, the number of influenza‐associated US adult hospitalizations was approximately 322,700 [10], and the estimated number of US adult hospitalizations for pneumococcal pneumonia ranged between 111,000 and 226,000 according to recent estimates [11, 12].

Study strengths include year‐round specimen collection, including from most patients hospitalized during months with increased RSV circulation, centralized molecular testing, and adjustment for patients without nasal samples available for RSV testing. Limitations include limited precision of some estimates due to the available sample size and estimates based on a single year during the COVID‐19 pandemic, and enrollment at one surveillance hospital, which required assumptions about the similarity of patients, healthcare seeking, and hospitalizations between the enrollment hospital and other hospitals that provided healthcare to residents of the catchment areas. Furthermore, our estimates may include patients transferred from other health institutions, including other hospitals and long‐term care facilities, but we did not have additional information to stratify our analyses by source of admission.

This prospective assessment demonstrated the burden of RSV‐associated hospitalizations among adults in Middle Tennessee during the year prior to RSV vaccine introduction, with the highest hospitalization rates among adults ≥ 60 years old.

## Author Contributions

**Carlos G. Grijalva:** writing – original draft, formal analysis, conceptualization, methodology. **Jesse O. Wrenn:** writing – review and editing, formal analysis, data curation, investigation. **Jonathan E. Schmitz:** writing – review and editing, formal analysis, data curation. **Karen F. Miller:** writing – review and editing, data curation, investigation. **Adrienne Baughman:** writing – review and editing, investigation, data curation. **Ian D. Jones:** writing – review and editing, investigation, data curation. **James D. Chappell:** writing – review and editing, investigation, formal analysis. **Natasha B. Halasa:** writing – review and editing, investigation, formal analysis. **Paul W. Blair:** writing – review and editing, investigation. **Yuwei Zhu:** writing – review and editing, formal analysis, data curation. **H. Keipp Talbot:** writing – review and editing, investigation. **Jonathan D. Casey:** writing – review and editing, investigation, data curation. **Fatimah S. Dawood:** writing – review and editing, methodology. **Diya Surie:** writing – review and editing, methodology. **Wesley H. Self:** writing – review and editing, funding acquisition, conceptualization, methodology.

## Disclosure

Investigators from CDC were involved in all aspects of the analysis, including the design and conduct of the activity, collection, management, analysis, and interpretation of the data; preparation, review, or approval of the manuscript; and decision to submit the manuscript for publication. The CDC had the right to control decisions about publication via the CDC publication clearance process. The findings and conclusions in this report are those of the authors and do not necessarily represent the official position of the Centers for Disease Control and Prevention.

## Conflicts of Interest

James D. Chappell, MD, PhD, has received research support from Merck and QuidelOrtho, outside of the submitted work. Jesse O. Wrenn reports grant support from the CDC during the conduct of the study and grant support from the NIH, VA, and AHRQ, outside of the submitted work. Natasha B. Halasa, MD, MPH, has received grant support from the CDC, Sanofi, and Quidel; reports a current investigator‐initiated grant from Merck; and serves on an advisory board for CSL Seqirus. H. Keipp Talbot, MD, MPH, has received research support from the CDC. Jonathan E. Schmitz reports grant support from the CDC, outside the submitted work. Carlos G. Grijalva, MD, MPH, reports research support from the CDC during the conduct of the study and research support from the CDC, NIH, AHRQ, and Syneos Health, outside of the submitted work; and has served on a Scientific Advisory Board for Merck and GSK, outside the submitted work. Yuwei Zhu reports grant support from the CDC during the conduct of the study. Jonathan D. Casey has received personal fees from Reprieve Cardiovascular, outside the submitted work. Wesley H. Self reports grant support from the CDC during the conduct of the study. No other potential conflicts of interest were disclosed.

## Supporting information


**Figure S1:** Frequency of testing for (Panel A) and detection of (Panel B) RSV‐associated hospitalizations at the surveillance hospital among residents of a defined 9‐county catchment area, October 2022 through September 2023.
**Table S1:** ICD‐10 discharge diagnosis codes used to denote a hospitalization with acute respiratory illness or exacerbation of a cardiopulmonary condition.
**Table S2:** Observed RSV detections and projected counts at the surveillance hospital. Counts are reported by age group, enrollment period, type of RSV testing, and presence of an acute respiratory illness (ARI) discharge diagnosis.
**Table S3:** Summary of RSV testing and detections at the surveillance hospital.
**Table S4:** Two‐by‐two contingency table contrasting RSV detections from clinical and research tests. This table includes 1202 patients who had both clinical and research RSV tests completed.
**Table S5:** Patient characteristics for the 49 observed RSV‐associated hospitalizations.
**Table S6:** Observed and projected RSV hospitalizations at the surveillance hospital. This table shows observed and projected RSV hospitalizations at the surveillance hospital and probability of RSV detection, by age group.
**Table S7:** Surveillance hospital market share. This table shows total all‐cause hospitalizations in 2022 from catchment area residents, from catchment area residents at the surveillance hospital, and probability of hospitalization at the surveillance hospital (market share), by age group. Summary estimates were derived from the Tennessee Hospital Discharge Data System.
**Table S8:** Catchment area population. This table shows midyear 2023 US Census population estimates* for the nine Tennessee counties included in the catchment area for incidence calculations.
**Table S9:** Summary of RSV‐associated hospitalization incidence rates. This table shows estimated incidence rates of RSV‐associated hospitalizations among adults in Middle Tennessee (nine counties), United States, October 2022 through September 2023.
**Table S10:** Summary of RSV‐associated hospitalizations detected through research testing and without ARI diagnoses.

## Data Availability

The authors have nothing to report.

## References

[1] M. Melgar , A. Britton , L. E. Roper , et al., “Use of Respiratory Syncytial Virus Vaccines in Older Adults: Recommendations of the Advisory Committee on Immunization Practices—United States, 2023,” MMWR. Morbidity and Mortality Weekly Report 72, no. 29 (2023): 793–801. 37471262 10.15585/mmwr.mm7229a4 PMC10360650

[2] A. Britton , L. E. Roper , C. N. Kotton , et al., “Use of Respiratory Syncytial Virus Vaccines in Adults Aged >/=60 Years: Updated Recommendations of the Advisory Committee on Immunization Practices—United States, 2024,” MMWR. Morbidity and Mortality Weekly Report 73, no. 32 (2024): 696–702. 39146277 10.15585/mmwr.mm7332e1

[3] D. Surie , W. H. Self , Y. Zhu , et al., “RSV Vaccine Effectiveness Against Hospitalization Among US Adults 60 Years and Older,” JAMA 332, no. 13 (2024): 1105–1107. 39230920 10.1001/jama.2024.15775 PMC11375516

[4] F. P. Havers , M. Whitaker , M. Melgar , et al., “Burden of Respiratory Syncytial Virus‐Associated Hospitalizations in US Adults, October 2016 to September 2023,” JAMA Network Open 7, no. 11 (2024): e2444756. 39535791 10.1001/jamanetworkopen.2024.44756 PMC11561688

[5] A. R. Branche , L. Saiman , E. E. Walsh , et al., “Incidence of Respiratory Syncytial Virus Infection Among Hospitalized Adults, 2017‐2020,” Clinical Infectious Diseases 74, no. 6 (2022): 1004–1011. 34244735 10.1093/cid/ciab595

[6] B. Efron and R. Tibshirani , “Bootstrap Methods for Standard Errors, Confidence Intervals, and Other Measures of Statistical Accuracy,” Statistical Science 1 (1986): 54–75.

[7] R. G. Newcombe , “MOVER‐R Confidence Intervals for Ratios and Products of Two Independently Estimated Quantities,” Statistical Methods in Medical Research 25, no. 5 (2016): 1774–1778. 24108267 10.1177/0962280213502144

[8] J. M. McLaughlin , F. Khan , E. Begier , D. L. Swerdlow , L. Jodar , and A. R. Falsey , “Rates of Medically Attended RSV Among US Adults: A Systematic Review and Meta‐Analysis,” Open Forum Infectious Diseases 9, no. 7 (2022): ofac300. 35873302 10.1093/ofid/ofac300 PMC9301578

[9] A. C. Howa , Y. Zhu , D. Wyatt , et al., “Estimating the Undetected Burden of Respiratory Syncytial Virus Hospitalizations in Adults Through Capture‐Recapture Methods,” Influenza and Other Respiratory Viruses 18, no. 5 (2024): e13299. 38700006 10.1111/irv.13299 PMC11066857

[10] Centers for Disease Control and Prevention (CDC) . “Preliminary Estimated Flu Disease Burden 2022–2023 Flu Season”. Available From: https://www.cdc.gov/flu‐burden/php/data‐vis/2022‐2023.html. Last accessed 04/26/2025.

[11] W. Self , C. Whitney , A. Baughman , et al., Incidence of Pneumococcal Pneumonia Hospitalizations Before and During the COVID‐19 Pandemic in Tennessee and Georgia: Results From the Pneumo Study. International Symposium on Pneumococci & Pneumococcal Diseases Toronto Canada (2022).

[12] J. Ramirez , S. Furmanek , T. R. Chandler , et al., “Epidemiology of Pneumococcal Pneumonia in Louisville, Kentucky, and Its Estimated Burden of Disease in the United States,” Microorganisms 11, no. 11 (2023): 2813. 38004825 10.3390/microorganisms11112813 PMC10673027

