# Supplementary material for: Incidence Rates of RSV‐Associated Hospitalizations Among Adults in Middle Tennessee, United States, October 2022 Through September 2023
Source: Influenza Other Respir Viruses. 2025 Aug 25;19(8):e70150. doi: 10.1111/irv.70150 (PMC12378065; doi:10.1111/irv.70150)
Supplement: Supplementary file 1 — Figure S1: Frequency of testing for (Panel A) and detection of (Panel B) RSV‐associated hospitalizations at the surveillance hospital among residents of a defined 9‐county catchment area, October 2022 through September 2023. Table S1: ICD‐10 discharge diagnosis codes used to denote a hospitalization with acute respiratory illness or exacerbation of a cardiopulmonary condition. Table S2: Observed RSV detections and projected counts at the surveillance hospital. Counts are reported by age group, enrollment period, type of RSV testing, and presence of an acute respiratory illness (ARI) discharge diagnosis. Table S3: Summary of RSV testing and detections at the surveillance hospital. Table S4: Two‐by‐two contingency table contrasting RSV detections from clinical and research tests. This table includes 1202 patients who had both clinical and research RSV tests completed. Table S5: Patient characteristics for the 49 observed RSV‐associated hospitalizations. Table S6: Observed and projected RSV hospitalizations at the surveillance hospital. This table shows observed and projected RSV hospitalizations at the surveillance hospital and probability of RSV detection, by age group. Table S7: Surveillance hospital market share. This table shows total all‐cause hospitalizations in 2022 from catchment area residents, from catchment area residents at the surveillance hospital, and probability of hospitalization at the surveillance hospital (market share), by age group. Summary estimates were derived from the Tennessee Hospital Discharge Data System. Table S8: Catchment area population. This table shows midyear 2023 US Census population estimates* for the nine Tennessee counties included in the catchment area for incidence calculations. Table S9: Summary of RSV‐associated hospitalization incidence rates. This table shows estimated incidence rates of RSV‐associated hospitalizations among adults in Middle Tennessee (nine counties), United States, October 2022 through September 2023. Ta [file IRV-19-e70150-s001.docx]

**Supporting Information**

The Investigating Respiratory Viruses in the Acutely Ill (IVY) Network. Incidence Rates of RSV-associated Hospitalizations among Adults in Middle Tennessee, United States, October 2022 through September 2023

This supporting information has been provided by the authors to give readers additional details about their work.

**Table of Contents**

[Figure S1. Frequency of testing for (panel A) and detection of (panel B) RSV-associated hospitalizations at the surveillance hospital among residents of a defined 9-county catchment area, October 2022 through September 2023. 2](#_Toc196309014)

[Table S1. ICD-10 discharge diagnosis codes used to denote a hospitalization with acute respiratory illness or exacerbation of a cardiopulmonary condition 3](#_Toc196309015)

[Table S2. RSV detections and projected counts at the surveillance hospital. 4](#_Toc196309016)

[Table S3. Summary of RSV testing and detections at the surveillance hospital 7](#_Toc196309017)

[Table S4. Two-by-two contingency table contrasting RSV detections from clinical and research tests. 8](#_Toc196309018)

[Table S5. Patient characteristics for the 49 observed RSV-associated hospitalizations. 9](#_Toc196309019)

[Table S6. Observed and projected RSV hospitalizations at the surveillance hospital. 10](#_Toc196309020)

[Table S7. Surveillance hospital market share. 11](#_Toc196309021)

[Table S8. Catchment area population. 12](#_Toc196309022)

[Table S9: Summary of RSV-associated hospitalization incidence rates. 13](#_Toc196309023)

[Table S10: Summary of RSV-associated hospitalizations detected through research testing and without ARI diagnoses 14](#_Toc196309024)

# Figure S1. Frequency of testing for (panel A) and detection of (panel B) RSV-associated hospitalizations at the surveillance hospital among residents of a defined 9-county catchment area, October 2022 through September 2023.

**
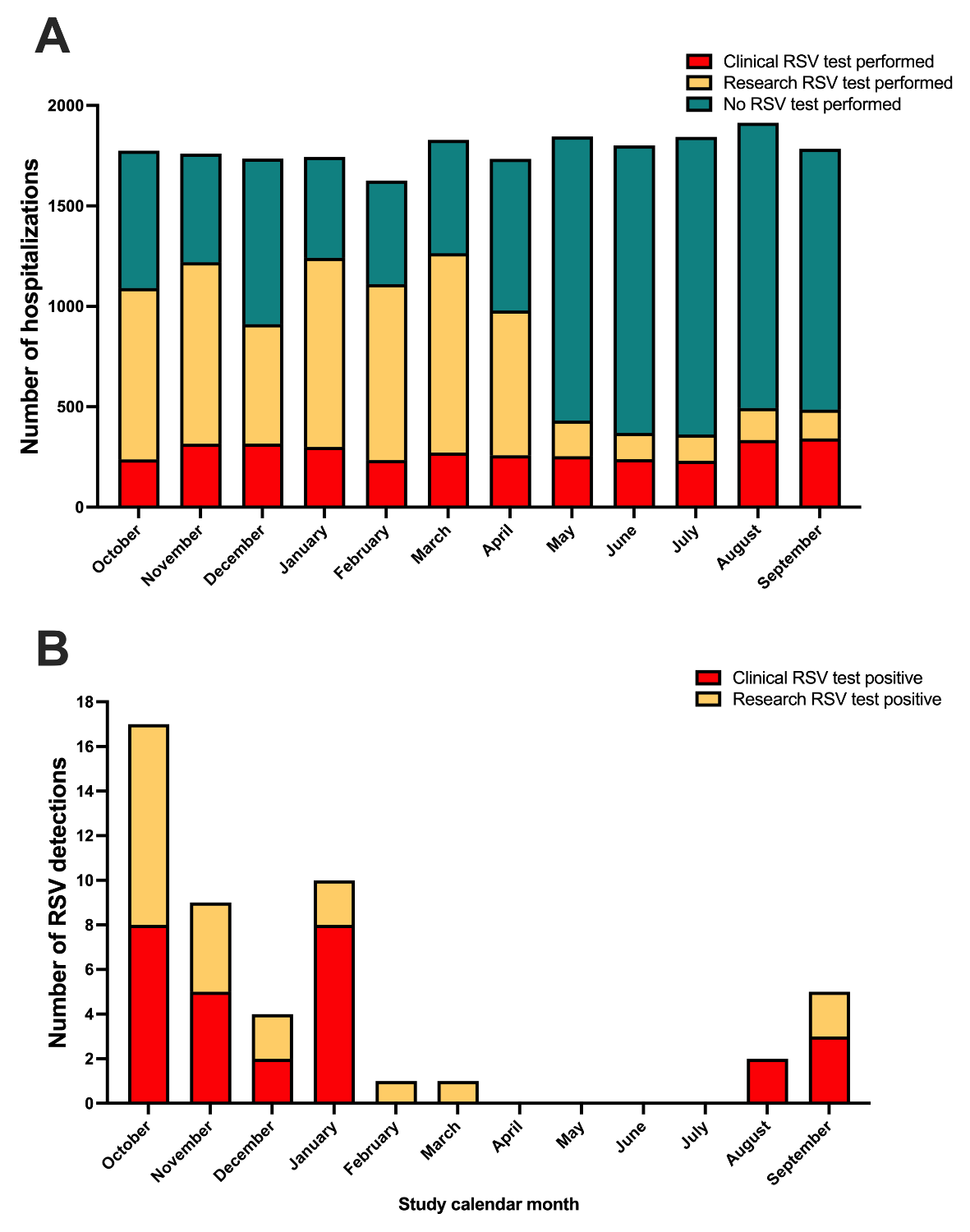
**

**Footnote:** Testing and detection groups were mutually exclusive. Hospitalizations with both clinical and research RSV testing performed were hierarchically classified as having clinical testing in these figures.

Table S1. ICD-10 discharge diagnosis codes used to denote a hospitalization with acute respiratory illness or exacerbation of a cardiopulmonary condition

| ICD-10 discharge diagnosis codes used to denote a hospitalization with acute respiratory illness or exacerbation of a cardiopulmonary condition |
| --- |
| J00, J01, J01.0, J01.00, J01.01, J01.1, J01.10, J01.11, J01.2, J01.20, J01.21, J01.3, J01.30, J01.31, J01.4, J01.40, J01.41, J01.8, J01.80, J01.81, J01.9, J01.90, J01.91, J02, J02.0, J02.8, J02.9, J03, J03.0, J03.00, J03.01, J03.8, J03.80, J03.81, J03.9, J03.90, J03.91, J04, J04.0, J04.1, J04.10, J04.11, J04.2, J04.3, J04.30, J04.31, J05, J05.0, J05.1, J05.10, J05.11, J06, J06.0, J06.9, J09, J09.X, J09.X1, J09.X2, J09.X3, J09.X9, J10, J10.0, J10.00, J10.01, J10.08, J10.1, J10.2, J10.8, J10.81, J10.82, J10.83, J10.89, J11, J11.0, J11.00, J11.08, J11.1, J11.2, J11.8, J11.81, J11.82, J11.83, J11.89, J12, J12.0, J12.1, J12.2, J12.3, J12.8, J12.81, J12.82, J12.89, J12.9, J13, J14, J15, J15.0, J15.1, J15.2, J15.20, J15.21, J15.211, J15.212, J15.29, J15.3, J15.4, J15.5, J15.6, J15.61, J15.69, J15.7, J15.8, J15.9, J16, J16.0, J16.8, J17, J18, J18.0, J18.1, J18.2, J18.8, J18.9, J20, J20.0, J20.1, J20.2,J20.3, J20.4, J20.5, J20.6, J20.7, J20.8, J20.9, J21, J21.0, J21.1, J21.8, J21.9, J22, J40, J80, J98.8, A22.1, A37.91, A37.01, A37.11, A37.81, A48.1, B25.0, B44.0, B97.4, O98.5, U07.1, U07.2, J45.2, J45.21, J45.22, J45.30, J45.31, J45.32, J45.40, J45.41, J45.42, J45.50, J45.51, J45.52, J45.901, J45.902, J45.909, J45.990, J45.991, J45.998, J82.83, J41.0, J41.1, J41.8, J42, J43.0, J43.1, J43.2, J43.8, J43.9, J44.0, J44.1, J44.9, I50.1, I50.20, I50.21, I50.22, I50.23, I50.30, I50.31, I50.32, I50.33, I50.40, I50.41, I50.42, I50.43, I50.810, I50.811, I50.812, I50.813, I50.814, I50.82, I50.83, I50.84, I50.89, I50.9, I09.81, I11.0, I13.0, I13.2 |

Table S2. Observed RSV detections and projected counts at the surveillance hospital. Counts are reported by age group, enrollment period, type of RSV testing, and presence of an acute respiratory illness (ARI) discharge diagnosis.

|  | **RSV**  **Negative** | **RSV**  **Positive** | **Total** | **Percent RSV Positive for projections** | **Observed and Projected Number of RSV Positives** |
| --- | --- | --- | --- | --- | --- |
| **18-49 years old**  **Oct 1 - Apr 11**  **(Screening for all admissions)** |  |  |  |  |  |
| Clinical test | 563 | 8 | 571 | 1.40 | 8 |
| Research test no ARI diagnosis | 1,836 | 5 | 1,841 | 0.27 | 5 |
| Research test ARI diagnosis | 123 | 0 | 123 | 0 | 0 |
| Not tested no ARI diagnosis | n/a | n/a | 1,291 | 0.27 | 4 |
| Not tested ARI diagnosis | n/a | n/a | 130 | 0 | 0 |
| Total |  | 13 | 3,956 |  | 17 |
| **18-49 years old**  **Apr 12 – Jul 31**  **(Screening for admissions with respiratory symptoms)** | | | | | |
| Clinical test | 277 | 0 | 277 | 0 | 0 |
| Research test no ARI diagnosis | 244 | 0 | 244 | 0 | 0 |
| Research test ARI diagnosis | 29 | 0 | 29 | 0 | 0 |
| Not tested no ARI diagnosis | n/a | n/a | 1,773 | 0 | 0 |
| Not tested ARI diagnosis | n/a | n/a | 84 | 0 | 0 |
| Total |  |  | 2,407 |  | 0 |
| **18-49 years old**  **Aug 1 – Sep 30**  **(Screening for admissions with respiratory symptoms)** | | | | | |
| Clinical test | 209 | 0 | 209 | 0 | 0 |
| Research test no ARI diagnosis | 81 | 0 | 81 | 0 | 0 |
| Research test ARI diagnosis | 9 | 0 | 9 | 0 | 0 |
| Not tested no ARI diagnosis | n/a | n/a | 975 | 0 | 0 |
| Not tested ARI diagnosis | n/a | n/a | 50 | 0 | 0 |
| Total |  |  | 1,324 |  | 0 |
| **50-59 years old**  **Oct 1 - Apr 11**  **(Screening for all admissions)** | | | | | |
| Clinical test | 294 | 8 | 302 | 2.65 | 8 |
| Research test no ARI diagnosis | 843 | 2 | 845 | 0.24 | 2 |
| Research test ARI diagnosis | 82 | 0 | 82 | 0 | 0 |
| Not tested no ARI diagnosis | n/a | n/a | 543 | 0.24 | 1 |
| Not tested ARI diagnosis | n/a | n/a | 95 | 0 | 0 |
| Total |  | 10 | 1,867 |  | 11 |
| **50-59 years old**  **Apr 12 – Jul 31**  **(Screening for admissions with respiratory symptoms)** | | | | | |
| Clinical test | 147 | 0 | 147 | 0 | 0 |
| Research test no ARI diagnosis | 113 | 0 | 113 | 0 | 0 |
| Research test ARI diagnosis | 19 | 0 | 19 | 0 | 0 |
| Not tested no ARI diagnosis | n/a | n/a | 707 | 0 | 0 |
| Not tested ARI diagnosis | n/a | n/a | 81 | 0 | 0 |
| Total |  |  | 1,067 |  | 0 |
| **50-59 years old**  **Aug 1 – Sep 30**  **(Screening for admissions with respiratory symptoms)** | | | | | |
| Clinical test | 106 | 0 | 106 | 0 | 0 |
| Research test no ARI diagnosis | 48 | 0 | 48 | 0 | 0 |
| Research test ARI diagnosis | 9 | 0 | 9 | 0 | 0 |
| Not tested no ARI diagnosis | n/a | n/a | 377 | 0 | 0 |
| Not tested ARI diagnosis | n/a | n/a | 53 | 0 | 0 |
| Total |  |  | 593 |  | 0 |
| **60-74 years old**  **Oct 1 - Apr 11**  **(Screening for all admissions)** | | | | | |
| Clinical test | 584 | 4 | 588 | 0.68 | 4 |
| Research test no ARI diagnosis | 1,401 | 4 | 1,405 | 0.28 | 4 |
| Research test ARI diagnosis | 257 | 0 | 257 | 0 | 0 |
| Not tested no ARI diagnosis | n/a | n/a | 887 | 0.28 | 3 |
| Not tested ARI diagnosis | n/a | n/a | 200 | 0 | 0 |
| Total |  | 8 | 3,337 |  | 11 |
| **60-74 years old**  **Apr 12 – Jul 31**  **(Screening for admissions with respiratory symptoms)** | | | | | |
| Clinical test | 310 | 0 | 310 | 0 | 0 |
| Research test no ARI diagnosis | 205 | 0 | 205 | 0 | 0 |
| Research test ARI diagnosis | 53 | 0 | 53 | 0 | 0 |
| Not tested no ARI diagnosis | n/a | n/a | 1,200 | 0 | 0 |
| Not tested ARI diagnosis | n/a | n/a | 205 | 0 | 0 |
| Total |  |  | 1,973 |  | 0 |
| **60-74 years old**  **Aug 1 – Sep 30**  **(Screening for admissions with respiratory symptoms)** | | | | | |
| Clinical test | 229 | 4 | 233 | 1.72 | 4 |
| Research test no ARI diagnosis | 80 | 1 | 81 | 1.23 | 1 |
| Research test ARI diagnosis | 18 | 1 | 19 | 5.26 | 1 |
| Not tested no ARI diagnosis | n/a | n/a | 693 | 1.23 | 9 |
| Not tested ARI diagnosis | n/a | n/a | 92 | 5.26 | 5 |
| Total |  | 6 | 1,118 |  | 20 |
| **75 or more years old**  **Oct 1 - Apr 11**  **(Screening for all admissions)** | | | | | |
| Clinical test | 306 | 7 | 313 | 2.24 | 7 |
| Research test no ARI diagnosis | 793 | 3 | 796 | 0.38 | 3 |
| Research test ARI diagnosis | 157 | 1 | 158 | 0.63 | 1 |
| Not tested no ARI diagnosis | n/a | n/a | 491 | 0.38 | 2 |
| Not tested ARI diagnosis | n/a | n/a | 164 | 0.63 | 1 |
| Total |  | 11 | 1,922 |  | 14 |
| **75 or more years old**  **Apr 12 – Jul 31**  **(Screening for admissions with respiratory symptoms)** | | | | | |
| Clinical test | 151 | 0 | 151 | 0 | 0 |
| Research test no ARI diagnosis | 119 | 0 | 119 | 0 | 0 |
| Research test ARI diagnosis | 30 | 0 | 30 | 0 | 0 |
| Not tested no ARI diagnosis | n/a | n/a | 736 | 0 | 0 |
| Not tested ARI diagnosis | n/a | n/a | 129 | 0 | 0 |
| Total |  |  | 1,165 |  | 0 |
| **75 or more years old**  **Aug 1 – Sep 30**  **(Screening for admissions with respiratory symptoms)** | | | | | |
| Clinical test | 127 | 1 | 128 | 0.78 | 1 |
| Research test no ARI diagnosis | 49 | 0 | 49 | 0 | 0 |
| Research test ARI diagnosis | 8 | 0 | 8 | 0 | 0 |
| Not tested no ARI diagnosis | n/a | n/a | 389 | 0 | 0 |
| Not tested ARI diagnosis | n/a | n/a | 89 | 0 | 0 |
| Total | 662 | 1 | 663 |  | 1 |

# Table S3. Summary of RSV testing and detections at the surveillance hospital

| **Type of RSV testing** | **Number of patients tested for RSV** | **Number of patients positive for RSV** | **Percentage of patients with a test positive for RSV** |
| --- | --- | --- | --- |
| Clinical test, including those with a research test | 3,335 | 32 | 0.96% |
| Clinical test, limited to patients without a research test | 2,133 | 21 | 0.98% |
| Research test, including those with a clinical test | 7,825 | 28 | 0.36% |
| Research test, limited to patients without a clinical test | 6,623 | 17 | 0.26% |
| Combination of clinical and research tests | 9,958 | 49 | 0.49% |

Table S4. Two-by-two contingency table contrasting RSV detections from clinical and research tests. This table includes 1,202 patients who had both clinical and research RSV tests completed.

|  |  | **Clinical RSV Test** | |
| --- | --- | --- | --- |
|  |  | ***Positive*** | ***Negative*** |
| **Research RSV test** | ***Positive*** | 4 (0.33%) | 4 (0.33%) |
|  | ***Negative*** | 3 (0.25%) | 1,191 (99.08%) |

# Table S5. Patient characteristics for the 49 observed RSV-associated hospitalizations.

| **Patient Characteristic** | | **RSV-associated hospitalizations (N=49)** |
| --- | --- | --- |
| Age, median (IQR) [years] | | 62.5 (47.0 to 74.9) |
| Race/ethnicity, n (%) | |  |
|  | White, not Hispanic | 31 (63.3) |
|  | Black, not Hispanic | 10 (20.4) |
|  | Hispanic | 4 (8.2) |
|  | Other or unknown | 4 (8.2) |
| Median hospital length of stay (IQR) [days] | | 5 (2 to 8) |
| In-hospital outcomes, n (%) | |  |
|  | ICU admission | 10 (20.4) |
|  | Invasive mechanical ventilation | 2 (4.1) |
|  | Death | 0 |

Table S6. Observed and projected RSV hospitalizations at the surveillance hospital. This table shows observed and projected RSV hospitalizations at the surveillance hospital and probability of RSV detection, by age group.

| **Age group** | **Total Observed RSV Cases** | **Total Estimated RSV Cases (observed plus projected cases)** | **Estimated Probability of RSV Detection (%)*** |
| --- | --- | --- | --- |
| 18-49 years old | 13 | 17 | 76.47 |
| 50-59 years old | 10 | 11 | 90.91 |
| 60-74 years old | 14 | 31 | 45.16 |
| 75 or more years old | 12 | 15 | 80.00 |
| All | 49 | 72 |  |

*The estimated probability of RSV detection was computed as the proportion of the total estimated number of RSV cases (including observed and projected RSV cases [Table S2]) that were observed as part of the study testing activities.

Table S7. Surveillance hospital market share. This table shows total all-cause hospitalizations in 2022 from catchment area residents, from catchment area residents at the surveillance hospital, and probability of hospitalization at the surveillance hospital (market share), by age group. Summary estimates were derived from the Tennessee Hospital Discharge Data System.

| **Age group** | **Hospitalizations among catchment areas residents at surveillance hospital** | **Total Hospitalizations among catchment residents overall** | **Probability of Hospitalization at surveillance hospital (i.e., market share)** |
| --- | --- | --- | --- |
| 18-49 years old | 10192 | 45195 | 0.2255 |
| 50-59 years old | 2506 | 15852 | 0.1581 |
| 60-74 years old | 3016 | 21576 | 0.1398 |
| 75 or more years old | 3705 | 36812 | 0.1006 |

Table S8. Catchment area population. This table shows mid-year 2023 US Census population estimates* for the 9 Tennessee counties included in the catchment area for incidence calculations.

| **County** | **18 to 49 years old** | **50 to 59 years old** | **60 to 74 years old** | **75 or more years old** | **Total Adults** |
| --- | --- | --- | --- | --- | --- |
| Cheatham | 16938 | 6124 | 7746 | 2475 | 33283 |
| Davidson | 358159 | 76302 | 96312 | 36224 | 566997 |
| Dickson | 22753 | 7735 | 9830 | 3687 | 44005 |
| Montgomery | 116516 | 23808 | 26930 | 8889 | 176143 |
| Robertson | 31136 | 10138 | 12750 | 4395 | 58419 |
| Rutherford | 172693 | 43478 | 45403 | 15624 | 277198 |
| Sumner | 84072 | 27683 | 34405 | 14476 | 160636 |
| Williamson | 102579 | 37786 | 40746 | 16192 | 197303 |
| Wilson | 67386 | 21440 | 26163 | 10695 | 125684 |
| Total | 972232 | 254494 | 300285 | 112657 | 1639668 |

*https://www.census.gov/data/tables/time-series/demo/popest/2020s-counties-detail.html

Table S9: Summary of RSV-associated hospitalization incidence rates. This table shows estimated incidence rates of RSV-associated hospitalizations among adults in Middle Tennessee (9 counties), United States, October 2022 through September 2023.

| **Age Group (years)** | **Detected RSV hospitalizations at surveillance hospital, n** | **Estimated proportion of RSV detection accounting for untested population, %** | **Market share of surveillance hospital for hospitalizations among catchment area population, %** | **Estimated RSV hospitalizations in catchment area (95% CI), n** | **Primary assessment for incidence of RSV-associated hospitalizations for catchment area [hospitalizations / 100,000 persons-year] (95% CI)** | **Incidence Rate ratio (95% CI)** | **Secondary assessment, accounting for imperfect sensitivity of RSV RT-PCR testing [hospitalizations / 100,000 persons-year] (95% CI)** |  |
| --- | --- | --- | --- | --- | --- | --- | --- | --- |
| 18–49 | 13 | 76.47 | 22.55 | 75 (61 to 89) | 7.71 (6.27 to 9.15) | Reference | 11.73 (9.46 to 13.89) | |
| 50–59 | 10 | 90.91 | 15.81 | 70 (62 to 85) | 27.51 (24.36 to 33.40) | 3.57 (2.89 to 4.74) | 41.26 (36.94 to 50.69) | |
| 60–74 | 14 | 45.16 | 13.98 | 222 (110 to 368) | 73.93 (36.63 to 122.55) | 9.59 (4.67 to 16.62) | 111.89 (55.61 to 185.82) | |
| ≥75 | 12 | 80.00 | 10.06 | 149 (126 to 179) | 132.26 (111.84 to 158.89) | 17.15 (13.50 to 22.61) | 200.61 (169.54 to 240.55) | |
| All adults | 49 |  |  | 516 (359 to 721) | 31.47 (21.89 to 43.97) |  | 47.69 (33.18 to 66.60) | |

Footnote: Estimated RSV hospitalizations were calculated as Detected RSV hospitalizations * (1 / (Estimated proportion of RSV detection [%] * proportion of admissions to the surveillance hospital [Market share %])).

# Table S10: Summary of RSV-associated hospitalizations detected through research testing and without ARI diagnoses

| **Age Group (years)** | **Hospital screening period** | **First listed discharge diagnosis** | **ICD10** |
| --- | --- | --- | --- |
| 18–49 | Screening for all admissions | Syphilis | A53.9 |
| 18–49 | Screening for all admissions | Hypoxia | R09.02 |
| 18–49 | Screening for all admissions | Clogged feeding tube | T85.598A |
| 18–49 | Screening for all admissions | Crohn's colitis, unspecified complication | K50.119 |
| 18–49 | Screening for all admissions | Vaginal bleeding | N93.9 |
| 50–59 | Screening for all admissions | Septic shock (CMS/HCC) | A41.9 |
| 50–59 | Screening for all admissions | Closed fracture of left distal tibia | S82.302A |
| 60–74 | Screening for admissions with respiratory symptoms | Pyelonephritis | N12 |
| 60–74 | Screening for all admissions | Syncope and collapse | R55 |
| 60–74 | Screening for all admissions | Volume overload | E87.70 |
| 60–74 | Screening for all admissions | Delirium | R41.0 |
| 60–74 | Screening for all admissions | Atrial fibrillation with rapid ventricular response | I48.91 |
| ≥75 | Screening for all admissions | Osteomyelitis | M86.9 |
| ≥75 | Screening for all admissions | Chest pain | R07.9 |
| ≥75 | Screening for all admissions | Esophagitis, erosive | K22.10 |
